# Aggressive lymphoma 2016: revision of the WHO classification

**DOI:** 10.1007/s12254-017-0367-8

**Published:** 2017-11-30

**Authors:** Christine Beham-Schmid

**Affiliations:** Institute of Pathology, Medical University Graz, Neue Stiftingtalstr. 6, 8010 Graz, Austria

**Keywords:** DLBCL, GCB, ABC, MYC, Double-hit

## Abstract

Aggressive lymphomas are a heterogeneous group of malignancies reflecting clinical, biological and pathological diversity. Diffuse large B‑cell lymphoma is the most common histological subtype and therefore will constitute the key aspect in this article. This lymphoma affects patients of all age groups with wide range presentations concerning localization, morphology and molecular mechanisms. The median age at presentation is about 60 years with a slight male preponderance. Up to 50% of patients present with advanced disease. About 70% of these lymphomas occur nodal, about 30% extranodal, the most common sites of the latter being the gastrointestinal tract, Waldeyer’s ring, skin, cerebrum, mediastinum, testis, salivary gland, thyroid and bone. However, diffuse large B‑cell lymphoma can involve virtually any organ.Since the last WHO classification 2008 the adoption of new genomic technologies has provided new insights into the biology of these lymphomas and led to the identification of distinct separate molecular entities and novel pathogenic pathways. These findings induced an expanding number of entities in the new WHO classification of 2016, the knowledge of which is essential concerning treatment options and survival of the patients. Therefore, the clinicians request an accurate diagnosis from the investigating pathologist, which can be quite challenging. The diagnosis of lymphomas requires multiple immunohistochemical studies, and often additional tests, such as fluorescent in situ hybridization and/or polymerase chain reaction techniques and occasionally, in particular cases, next generation sequencing for identification of recurrent somatic mutations. This review summarizes relevant aspects of the new WHO classification in aggressive B‑cell lymphomas, especially from a haematopathologist’s point of view.

## Introduction

Aggressive B‑cell lymphomas consist of precursor lymphoid neoplasms (B-lymphoblastic leukaemia/lymphoma NOS and B‑lymphoblastic leukaemia/lymphoma with recurrent genetic abnormalities) and numerous mature B‑cell neoplasms, such as mantle cell lymphoma, Burkitt lymphoma, primary effusion lymphoma, diffuse large B‑cell lymphoma (DLBCL) with its numerous subtypes, B‑cell lymphoma unclassifiable with features between DLBCL and Burkitt lymphoma, and the new variant Burkitt-like lymphoma with 11q aberrations (see Table 1).

**Table 1:** WHO classification of mature large B‑cell lymphoid neoplasms

Diffuse large B‑cell lymphoma (DLBCL), NOS – Germinal centre B‑cell type^a^ – Activated B‑cell type^a^
T-cell/histiocyte-rich large B‑cell lymphoma
Primary DLBCL of the central nervous system (CNS)
EBV+ DLBCL, NOS^a^
*EBV+ mucocutaneous ulcer* ^a^
DLBCL associated with chronic inflammation
Lymphomatoid granulomatosis
Primary mediastinal/thymic large B‑cell lymphoma
Intravascular large B‑cell lymphoma
ALK+ large B‑cell lymphoma
Plasmablastic lymphoma
Primary effusion lymphoma
*HHV8+ DLBCL, NO* *S* ^a^
Burkitt lymphoma
*Burkitt-like lymphoma with 11q aberration* ^a^
High grade B‑cell lymphoma, with MYC and BCL2 and/or BCL6 rearrangements^a^
High grade B‑cell lymphoma, NOS^a^
B-cell lymphoma, unclassifiable, with features intermediate between DLBCL and classical Hodgkin lymphoma

The provisional entities are listed in italics

^a^Changes from the 2008 classification

*NOS* not otherwise specified, *EBV* Epstein Barr virus

Diffuse large B‑cell lymphoma (DLBCL) is an aggressive B‑cell lymphoma histologically characterized by dense proliferation of neoplastic B‑blasts. DLBCL is the most common histological subtype of non-Hodgkin lymphomas (NHL) accounting worldwide for about 30% of adult NHL [1]. Within the group of DLBCL, many disparate entities with enormous differences concerning the clinical behaviour, morphology, immunophenotype and molecular mechanisms are known. Since the response to treatment regimens is different in the subtypes of DLBCLs, intensive research has specialized on the identification of prognostically or biologically distinct groups aiming at tailored treatment regimens. Thus, prognostic markers are of great importance [2]. The WHO classification 2008 [3] already recognized the importance of the cell-of-origin classification into germinal centre B‑cell-like (GCB) and activated B‑cell-like (ABC) molecular subgroups of DLBCLs not otherwise specified (NOS) based on gene expression profiling (GEP). In addition a group of cases that could not be put into either category (unclassifiable) was found. The GEP-defined GCB and ABC subtypes are different concerning chromosomal alterations, activation of signaling pathways and clinical outcome [4]. Since the estimation of GEP-defined subgroups requires fresh or frozen tissue for extraction of sufficient RNA, which is difficult to implement in clinical practice, the use of immunohistochemistry with special antibodies, which represent different stages of B‑cell differentiation, has been proposed. Various immunohistochemical algorithms predicting the prognosis of DLBCL and guiding treatment options have been reported [5, 6], revealing the Choi algorithm as the most predictive of GEP results [7]. Accumulated knowledge of the molecular pathogenesis of these 2 subgroups resulted in the investigation of more specific therapeutic strategies to appease the poorer outcome among those with ABC (non-GCB) type DLBCL reported in most studies [8]. The revised WHO classification 2016 now requires the cell of origin identification.

In addition, the WHO update recognizes immunohistochemical coexpression of MYC and BCL2 proteins within DLBCL NOS as a new adverse prognostic marker, but not a separate category. These DLBCL NOS are called “double-expressor lymphomas” and are strongly associated with poor outcome in patients treated with R‑CHOP. According to Green et al. [9] a double-hit score (DHS) can be assigned to all patients with DLBCL.

A worse outcome than double-expressor lymphomas show high grade B‑cell lymphomas (HGBL) with rearrangements of MYC and BCL2 and/or BCL6, the so-called double-hit or triple-hit lymphomas, which constitute a single category in the updated WHO classification.

## Primary DLBC in central nervous system, testis and stomach

**Fig. 1:**
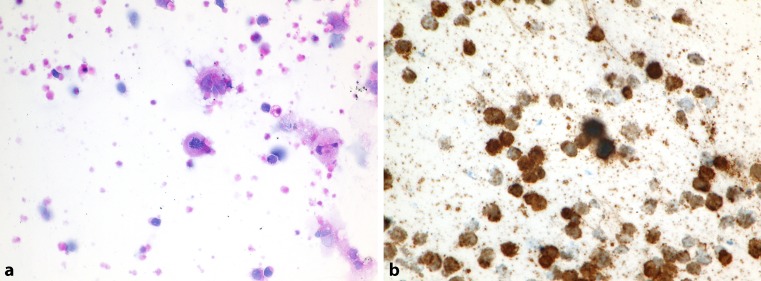
Cytology sample (vitreous preparation) with infiltration by ocular diffuse large B‑cell lymphoma (DLBCL). **a** H&E; **b** Tumour cells stained with an antibody to CD20

Approximately 95% of primary central nervous system lymphomas (PCNSL) are large B‑cell lymphomas. They reveal a unique pathobiology with dissemination of the lymphoma within the brain, cranial nerves, leptomeninges, cerebrospinal fluid, intraocular structures (Fig. 1) and spinal cord, without overt systemic disease. Most of PCNSL cases are of an activated B‑cell immunophenotype [10]. Since the brain is largely assumed to be an immunologically quiescent or “privileged site”, PCNSL require different treatment options [11].

Primary lymphomas of the testis with morphology of DLBCL in most cases are of an activated B‑cell immunophenotype. The testis is also considered a “privileged” localization with special treatment regimens [12].

Treatment of primary gastric diffuse large B‑cell lymphoma is controversial; however, regression of high-grade gastric lymphomas after the cure of Helicobacter pylori infection have been described [13].

The clinical differences between nodal and extranodal DLBCL thus require the distinction between nodal and extranodal DLBCL.

## Cell of origin classification

Within the large category of DLBCL NOS two molecular subtypes have been recognized by GEP, termed germinal centre B‑cell (GCB) and activated B‑cell (ABC) with a small proportion of patients (about 15%) remaining unclassifiable. The ABC subtype shows a worse outcome with R‑CHOP treatment [8]. These subtypes are provoked by various different oncogenic pathways which might be important for therapeutic benefits [14].

### GCB DLBCL

GCB DLBCL derives from centroblasts and thus express genes which can be detected in germinal centre B‑cells, mainly GCET1, CD10 and BCL6. About one third of GCB DLBCL reveal a c-rel amplification or a t(14;18) translocation and 10–20% show mutations of the histone methyltransferase EZH2 or a deletion of PTEN. These abnormalities are not found in ABC subtypes. PTEN, which is an essential tumour suppressor gene encoding a phosphatase protein that antagonises the PI3K/Akt/mTOR antiapoptotic pathway, might be susceptible to new treatment options [15].

Different oncogenic pathways give rise to an overexpression of BCL2, an antiapoptotic protein, in GCB and also ABC DLBCL. Whereas BCL2 overexpression in GCB subtype is caused by t(14;18) translocation, in ABC subtype gene amplification and transcriptional upregulation provokes BCL2 overexpression. Appliance of BCL2 inhibitors in DLBCL is an auspicious therapy option [16].

### ABC DLBCL

ABC DLBCL originates from plasmablastic cells prior to germinal centre exit and shows a gene expression similar to that of mature plasma cells, like MUM1and FOXP1. Typical for ABC DLBCL is the constitutive activation of the NF-kappaB signalling pathway which is essential for cell survival and proliferation and inhibition of apoptosis. More than 50% of ABC DLBCL show mutations concerning the regulation of the NF-kappaB cascade: Activating mutations of CARD11are found in 10% of ABC subtype, 20% show mutations in CD79A or CD79B, and about 30% reveal recurring mutations in MYD88.

### Identification of the cell of origin

**Fig. 2:**
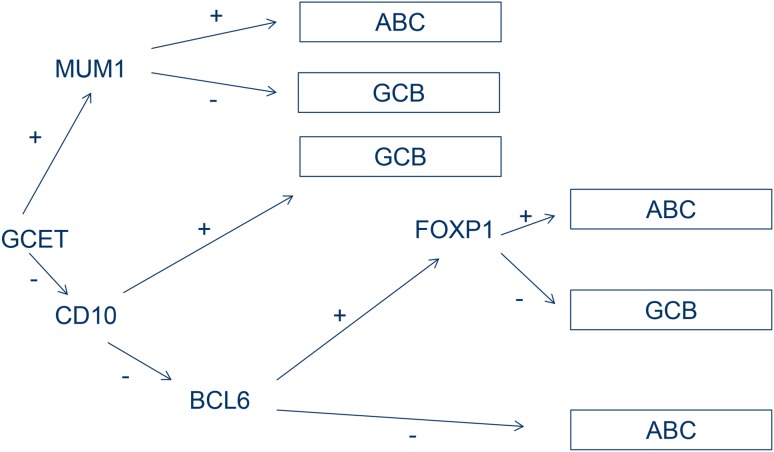
Schematic representation of immunohistochemical algorithm according to Choi et al. [6]

**Fig. 3:**
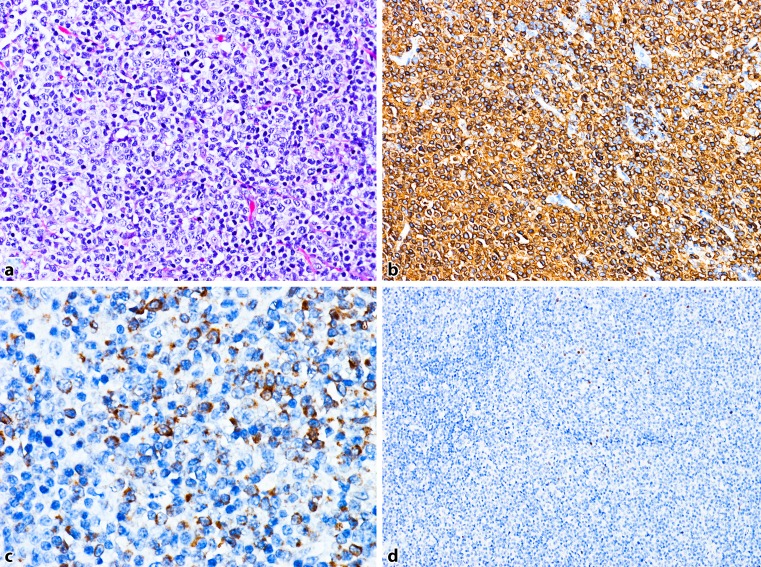
DLBCL, GCB type: **a** dense lymph node infiltration by lymphatic blasts (H&E). **b** The blasts are strongly CD20-positive. **c** Many tumour cells are GCET1-positive. **d** No reactivity with an antibody to MUM1

**Fig. 4:**
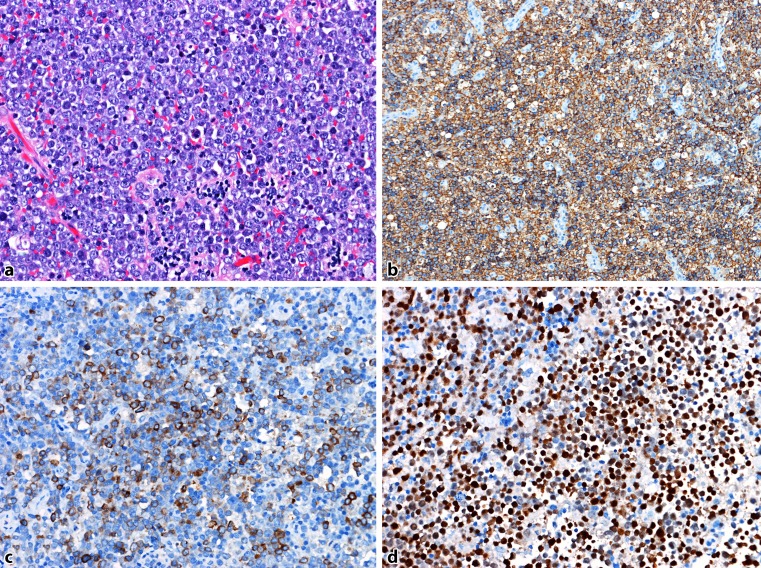
DLBCL, ABC type: **a** dense lymph node infiltration by lymphatic blasts (H&E). **b** Strong CD20-positivity in the tumour cells. **c** Many tumour cells are GCET1-positive. **d** Strong nuclear expression of MUM1

Since GEP is not routinely available, a reliable method of determining the molecular subtype is the use of immunohistochemically (IHC) based algorithms. Since the first published Hans algorithm in 2004 [5], which uses three markers (CD10, BCL6 and MUM1), several improved IHC algorithms have been developed. The WHO classification does not specify which method should be used. The algorithm developed by Choi et al. ([6]; Fig. 2) is consistent in 93% with results of GEP. IHC algorithms are easily applicable and are a reliable way for the evaluation of GCB or ABC subtype (Figs. 3 and 4).

Recent techniques using NanoString technology on paraffin embedded tissue are possibly the future way for estimation of GCB or ABC subtype [17].

## Role of the microenvironment and GEP

Concerning the outcome of DLBCL patients GEP studies revealed molecular signatures in the microenvironment of DLBCL NOS. High numbers of macrophages and extracellular matrix deposition (stromal-1 signature) is associated with a better prognosis than stromal-2 signature with marked angiogenesis. Both signatures could be detected in GCB and ABC subtypes. Furthermore, genetic aberrations concerning antigen presenting functions and immune recognition have been identified, probably providing therapeutic possibilities [18].

## Importance of BCL2 and MYC

**Fig. 5:**
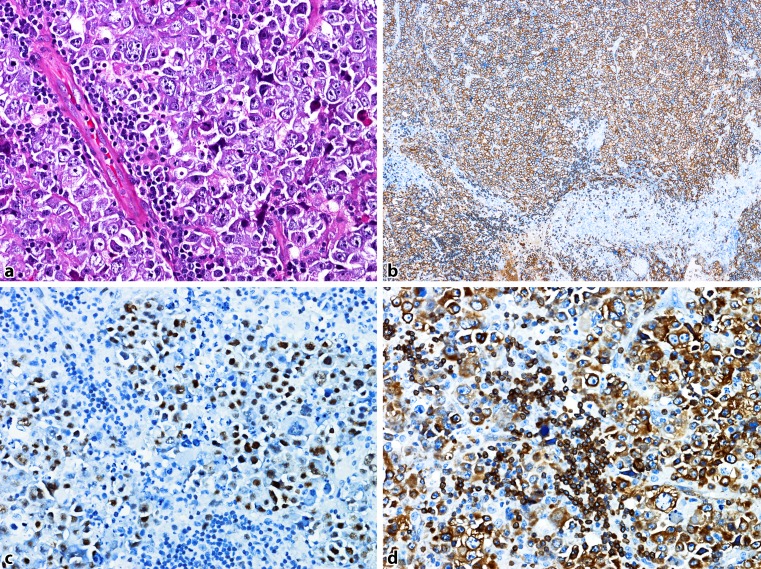
DLBCL, “double-expressor”: **a** Dense infiltration of a lymph node by lymphatic blasts (H&E). **b** Staining with an antibody to CD20 revealing B‑cell origin. **c** More than 40% of the blasts reveal nuclear MYC-positivity. **d** The blasts are strongly BCL2-positive

MYC rearrangement, typically detected by fluorescence in situ hybridization (FISH), is characteristic for Burkitt lymphoma. However, in about 10% of DLBCL NOS, rearrangement of MYC is found and has been shown to be associated with a poor outcome in patients treated with R‑CHOP. Frequently, MYC rearrangement is combined with BCL2, and/or less frequent with BCL6 translocation. These “double-hit” or “triple-hit” lymphomas [19] are included in the updated WHO classification in the category of high-grade B‑cell lymphoma (HGBL), with rearrangements of MYC and BCL2 and/or BCL6. Surprisingly, most of these cases are of GCB type, which, accordingly to the cell of origin classification, usually show a better prognosis than ABC types with R‑CHOP treatment. Routinely, the status of MYC, BCL2 and BCL6 is analyzed by FISH techniques. An immunohistochemical overexpression of MYC is found more frequently than MYC translocation. Up to 30% of patients with DLBCL overexpress MYC protein. A poor prognostic impact of patients with MYC protein expression is found when BCL2 protein is coexpressed (Fig. 5). These so-called “double-expressors” [9], seen in up to 25% of DLBCL patients, are an independent predictor of poor survival, and might be important to stratify patients for risk-adapted therapies. Immunohistochemical evaluation of MYC, BCL2 and BCL6 has been extensively discussed [20]. In most studies a DLBCL is considered MYC positive when ≥40% of blasts show MYC expression. The cutoff for BCL2 positivity is rather discordant with values ranging from 30 to 70% as considered being positive [21].

## Burkitt-like lymphoma with 11q aberration

For a long time it was a matter of debate whether true Burkitt lymphoma (BL) without *MYC* translocation does exist. Many studies reported lymphomas with clinical, histologic, immunophenotypic, or gene expression features of BL, but no detectable *MYC* translocation by FISH analysis. One should be extremely cautious to diagnose a true *MYC*-negative BL because the scattering of breakpoints in the *MYC* and *IG* loci along with small insertions of one locus into the other can render *MYC* breaks undetectable even if several sets of FISH probes are applied. Salaverria et al. [22] described a subset of lymphomas with gene expression and pathological characteristics of Burkitt lymphomas but absence of *MYC* translocation. These lymphomas carry chr 11q proximal gains and telomeric losses, suggesting coderegulation of oncogenes and tumour suppressor genes. Although only a small number of these lymphomas have been reported, the clinical course is similar to that of BL.

## Conclusion

Risk stratification of the heterogeneous group of DLBCL NOS is an evolving procedure. Ongoing efforts to tailor therapy based on the different oncogenic pathways of GCB and ABC DLBCL demand an optimal cell of origin classification. Furthermore, the detection of MYC gene translocation and MYC protein expression has shown to be of increasing importance in the prognosis and treatment of DLBCL patients. It is recommended to investigate for both dual or trifold translocation (MYC, BCL2 and BCL6) and dual protein expression status, since treatment options may be different. It is important to mention that immunohistochemical MYC protein expression does not equate to MYC rearrangement. However, a correlation has been shown between immunohistochemical MYC protein expression and MYC gene abnormalities in DLBCL [23]. Routinely, the diagnosis of DLBCL NOS includes the cell of origin classification and the double-hit score (“double-expressor”) according to Green et al. [9]. The next step, if MYC-protein expression has been detected, is FISH investigation for MYC, and the partner genes BCL2 and BCL6. The results are reported subsequently.

To identify relevant disease drivers that are therapeutically targetable, concerted efforts generated the so-called “lymphopanel”. This targeted gene sequencing panel enables the detection of mutations and subtype-enriched gene alterations in DLBCL hopefully yielding to the development of new and effective targeted treatment approaches [24].

### Take home message

Diagnosis of DLBCL must include cell of origin classification (GCB, ABC) and immunohistochemical double hit score (MYC, BCL2).

Coexpression of MYC and BCL2 is considered a new prognostic marker.

The understanding of the mutational landscape might become part of the foundation for optimal treatment options.
